# LiCl treatment leads to long-term restoration of spine maturation and synaptogenesis in adult *Tbr1* mutants

**DOI:** 10.1186/s11689-022-09421-5

**Published:** 2022-02-05

**Authors:** Siavash Fazel Darbandi, Andrew D. Nelson, Emily Ling-lin Pai, Kevin J. Bender, John L. R. Rubenstein

**Affiliations:** 1 Department of Psychiatry and Behavioral Sciences, and Weill Institute for Neurosciences, University of California San Francisco, San Francisco, CA USA; 2 Weill Institute for Neurosciences, University of California, San Francisco, CA USA; 3 Department of Neurology, University of California, San Francisco, CA USA

**Keywords:** Autism spectrum disorder, *Tbr1*, Dendritic spine, Synaptogenesis, Excitatory neuron, Cortex, Synaptic transmission, Synaptic rescue, mPFCx

## Abstract

**Background:**

*Tbr1* encodes a T-box transcription factor and is considered a high confidence autism spectrum disorder (ASD) gene. *Tbr1* is expressed in the postmitotic excitatory neurons of the deep neocortical layers 5 and 6. Postnatally and neonatally, *Tbr1* conditional mutants (CKOs) have immature dendritic spines and reduced synaptic density. However, an understanding of *Tbr1*’s function in the adult mouse brain remains elusive.

**Methods:**

We used conditional mutagenesis to interrogate *Tbr1*’s function in cortical layers 5 and 6 of the adult mouse cortex.

**Results:**

Adult *Tbr1* CKO mutants have dendritic spine and synaptic deficits as well as reduced frequency of mEPSCs and mIPSCs. LiCl, a WNT signaling agonist, robustly rescues the dendritic spine maturation, synaptic defects, and excitatory and inhibitory synaptic transmission deficits.

**Conclusions:**

LiCl treatment could be used as a therapeutic approach for some cases of ASD with deficits in synaptic transmission.

**Supplementary Information:**

The online version contains supplementary material available at 10.1186/s11689-022-09421-5.

## Introduction

Genetic heterogeneity and phenotypic pleiotropy have complicated efforts in understanding the underlying biology of autism spectrum disorder (ASD) [1, 2]. Whole-genome and exome sequencing has revealed 102 high confidence ASD (hcASD) risk genes (FDR < 0.1) [3]. Systems analyses suggest that the expression of ASD risk genes have important functions in mid-fetal deep layer cortical excitatory neurons and that disruption may contribute to ASD pathophysiology [2]. The hcASD-risk genes fall into two major categories of gene expression regulators and synaptic genes [1, 3, 4]. However, an actionable understanding of whether ASD-risk gene expression regulators control and/or contribute to synaptic pathophysiology remains unclear.

Genetic analyses of patients with ASD have identified TBR1 as a high confidence risk factor for ASDs [2, 4–6]. Analyses of co-expression networks of ASD-risk genes provide evidence that reduced dosage of genes, such as *Tbr1*, may underlie ASD by disrupting processes in immature projection neurons of deep cortical layers during human mid-fetal development [2]. To date, there are 5 de novo loss-of-function mutations in *TBR1* that are associated with ASD [7]. Two of the *TBR1* de novo mutations generate truncated proteins that lack a functional DNA-binding T-box domain [4]. These two truncated TBR1 mutants lose their ability to regulate transcription, have an altered intracellular distribution, and fail to interact with other proteins including CASK [8, 9] and FOXP2 [7]. Thus, analysis of the *Tbr1* transcription factor (TF) has opened the possibility of defining a transcriptional pathway that contributes to ASD pathology [5, 6].

*Tbr1* has a central role in the development of mouse early-born excitatory cortical neurons. *Tbr1* expression, which begins in newborn neurons, dictates layer 6 identity by repressing *Fezf2* and *Bcl11b*, transcription factors that control layer 5 fate [10–14]. We previously demonstrated that young adult (P56) *Tbr1*^*layer5*^ and *Tbr1*^*layer6*^ CKO mutants have reduced dendritic spine density as well as reduced excitatory and inhibitory synaptic density [15, 16]. Furthermore, we demonstrated that *Tbr1* mutant layer 5 and 6 cortical neurons have reduced WNT signaling that underlies their defects in dendritic spines and synaptic density. Consistent with this, lithium chloride (LiCl) treatment, or inhibition of GSK3β, rescued these defects [15]. LiCl is an FDA-approved drug that promotes WNT signaling [17–19].

Towards evaluating whether LiCl rescue could be relevant for the treatment of human *TBR1* mutant patients, we evaluated whether *Tbr1* mutant mice have (1) dendritic spine and synaptic deficits that persist into adulthood, (2) whether LiCl treatment can rescue the phenotype in the older animals, and (3) whether LiCl treatment rescued synaptic transmission. Here, we demonstrate that adult *Tbr1*^*layer5*^ and *Tbr1*^*layer6*^ CKO mutants have dendritic spine and synaptic density deficits. Furthermore, adult *Tbr1* CKO mutants have reduced mEPSC and mIPSC frequencies. Furthermore, we have demonstrated that the LiCl rescue of the spine and synaptic density in the *Tbr1*^*layer5*^ and *Tbr1*^*layer6*^ CKO mutants is sustained 6 months after the LiCl injection, which indicates the long-term efficacy of LiCl treatment on rescuing spine and synaptic density deficits. Lastly, we show that LiCl rescues the spine and synaptic defects in adult *Tbr1* CKOs, which may have therapeutic implications for ASD patients with either mutations in *TBR1* or for cases with impaired synaptic function.

## Materials and methods

### Animals

All procedures and animal care were approved and performed in accordance with the University of California San Francisco Laboratory Animal Research Center (LARC) guidelines (Animal protocol # AN180174-03B). All strains were maintained on a C57Bl/6 background. Animals were housed in a vivarium with a 12-h light (7 am to 7 pm), 12-h dark cycle. Animals were tested during the light cycle. Experimental animals were kept with their littermates.

The *Tbr1*^*flox*^ allele was generated by the inGenious Targeting Laboratory (Ronkonkoma, NY). LoxP sites were inserted into introns 1 and 3, flanking *Tbr1* exons 2 and 3 [16]. To enable the selection of homologous recombinants, the LoxP site in intron 3 was embedded in a *neo* cassette that was flanked by *Flp* sites. The *neo* cassette was removed by mating to a *Flp*-expressing mouse to generate the *Tbr1*^*flox*^ allele. Cre excision removes exons 2 and 3, including the T-box DNA binding region, similar to the constitutive null allele [13]. *Rbp4-cre* (Gensat KL100) and *Ntsr1-cre* (Gensat 220) mice were used to delete *Tbr1* in layer 5 and layer 6 projection neurons, respectively. *tdTomato*^*fl/+*^ (*Ai14*) mice were crossed with *Tbr1*^*f/f*^ mice and used as an endogenous reporter. *Tbr1* layer 5 and layer 6 conditional knockout mice were generated as previously described [15, 16].

### Transgenic animal models

The mouse strains used for this research project, B6.FVB (Cg)-Tg (Ntsr1-cre)GN220Gsat/Mmucd, RRID:MMRRC_030648-UCD, and B6.FVB (Cg)-Tg (Rbp4-cre)KL100Gsat/Mmucd, RRID:MMRRC_037128-UCD were obtained from the Mutant Mouse Resource and Research Center (MMRRC) at the University of California at Davis, an NIH-funded strain repository, and was donated to the MMRRC by MMRRC at UCD, University of California, Davis. Made from the original strain (MMRRC:032081) donated by Nathaniel Heintz, Ph.D., The Rockefeller University, GENSAT https://protect2.fireeye.com/url?k=c19cddd3-9ddce8ed-c19cface-0cc47ad9c120-2678b1e782f452c7&u=http://www.gensat.org/ and Charles Gerfen, Ph.D., National Institutes of Health, National Institute of Mental Health.

Information about the generation and genotyping of the transgenic lines used in this study can be found in the corresponding original studies: *Rbp4-Cre* [20], lox-STOP-lox-tdTomato (Ai14; [21]). Mice were maintained on C57BL/6J background.

### Genomic DNA extraction and genotyping

Tail clippings were digested in a solution containing 1 mg/mL of proteinase K, 50 mM Tris-HCl pH 8.0, 100 mM EDTA, 100 mM NaCl, and 1% SDS. Genomic DNA was extracted using a standard ethanol precipitation protocol. Genotyping was performed with PCR-based assays using purified genomic DNA and primer-pair combinations flanking the deleted region and detecting *Cre* and *tdTomato* alleles.

### In vivo synapse rescue assay

We performed in vivo rescue assay of synaptic deficit in *Tbr1* mutant mice by using a single intraperitoneal (IP) injection of LiCl to rescue the decrease in synapse numbers in *Tbr1* mutants. P180 mice were administered a single IP injection of 400 mg/kg LiCl or saline in a volume of 4 ml/kg [18]. A separate cohort of P30 mice was administered a single IP injection of 400 mg/kg LiCl or saline in a volume of 4 ml/kg. Treated mice were anesthetized and perfused 6 months after LiCl injection, as described below.

### Histology

Animals were anesthetized with an intraperitoneal injection of 100 mg/kg ketamine containing 15 mg/kg xylazine. Animals were then perfused transcardially with ice-cold 1× PBS and then with 4% PFA in 1× PBS, followed by brain isolation, 1–2 h post-fixation in 4% PFA in 1X PBS at RT, cryoprotected in 30% sucrose in 1× PBS overnight at 4 °C, and cut frozen coronally on a sliding microtome at 40 μm for immunohistochemistry. All primary and secondary antibodies were diluted in 1× PBS containing 10% normal serum, 0.25% Triton X-100, and 2% BSA. The following primary antibodies were used: mouse anti-Vglut1 (1:200, Synaptic Systems; RRID: AB_887875), rabbit anti-Vgat (1:500, Synaptic Systems; RRID: AB_887871), rabbit anti-PSD95 (1:200, Cell Signaling; RRID: AB_2307331), and mouse anti-gephyrin (1:200, Synaptic Systems; RRID: AB_887717). The secondary antibodies for immunofluorescence were goat anti-rabbit IgG Alexa Fluor 488 (1:1000, Thermo Fisher; RRID: AB_143165) and goat anti-mouse Alexa Fluor 647 (1:1000, Thermo Fisher; RRID: AB_2633277). For in vivo synapse immunohistochemistry, a total of *n* = 10 apical dendrites were counted from each of *Tbr1*^*wildtype*^ and *Tbr1*^*layer5*^ homozygous mutants of mixed gender. The coronal sections were pre-treated with pepsin to enhance the staining. Immunofluorescence specimens were counterstained with 1% DAPI to assist the delineation of cortical layers.

### Image acquisition and analysis

Fluorescent and bright-field images were taken using a Coolsnap camera (Photometrics) mounted on a Nikon Eclipse 80i microscope using the NIS Elements acquisition software (Nikon). Confocal imaging experiments were conducted at the Cancer Research Laboratory (CRL) Molecular Imaging Center, supported by Helen Wills Neuroscience Institute at UC Berkeley. Confocal images were acquired using Zeiss LSM 880 with Airyscan with a 63X oil objective at 1024 × 1024 pixels resolution with 2.0X optical zoom using the ZEN 2.0 software. The 2.0X optical zoom allowed a higher magnification to visualize the dendritic spines prior to imaging. Brightness and contrast were adjusted, and images merged using the Fiji ImageJ software. The Fiji ImageJ software was used for image processing [22, 23]. For synapse counting (presynaptic and postsynaptic boutons), confocal image stacks (0.4μm step size) were processed with the Fiji ImageJ software. In brief, background subtraction and smooth filter were applied to each stack. Using a threshold function, each stack was converted into a “mask” image. Furthermore, the channels were co-localized with the Image Calculator plugging. Lastly, the number of co-localizations were counted, and the length of each dendrite was measured in each of the focal plane. Staining for control and mutant were done in parallel as well as the image capturing.

### Ex vivo electrophysiology

*Tbr1* ^*layer5*^ *::Rbp4-Cre::tdTomato* and *Tbr1*^*layer6*^*::Ntsr1-Cre::tdTomato* homozygous conditional knockout mice and wild-type littermates were administered a single, intraperitoneal (IP) injection of 400 mg/kg LiCl or 4 ml/kg of saline at P30. Mice aged P70-110 were anesthetized under isoflurane. Brains were dissected and placed in 4 °C cutting solution consisting of (in mM) 87 NaCl, 25 NaHCO_3_, 25 glucose, 75 sucrose, 2.5 KCl, 1.25 NaH_2_PO_4_, 0.5 CaCl_2_, and 7 MgCl_2_ and bubbled with 5% CO_2_/95% O_2_. Coronal slices 250-μm-thick were obtained that included the medial prefrontal cortex or the somatosensory cortex. Slices were then incubated in a holding chamber with sucrose solution for 30 mins at 33 °C, then placed at room temperature until recording. External aCSF recording solution consisted of (in mM): 125 NaCl, 2.5 KCl, 2 CaCl_2_, 1 MgCl_2_, 25 NaHCO_3_, 1.25 NaH_2_PO_4_, and 25 glucose, approximately 310 mOsm, bubbled with 5% CO_2_/95% O_2_, and maintained at 32–34 °C. Miniature excitatory and inhibitory postsynaptic currents (mEPSCs, mIPSCs) were measured in the presence of 10 μM R-CPP and 400 nM TTX. *td-Tomato*-positive neurons were identified using a Rolera-XR camera and conventional whole-cell patch clamp was conducted under differential interference contrast (DIC) optics.

mEPSC and mIPSC recordings were performed in voltage-clamp using glass patch electrodes (Schott 8250, 3–4 MΩ tip resistance) filled with a CsCl-based internal solution that contained (in mM): 110 CsMeSO_3_, 40 HEPES, 1 KCl, 4 CaCl, 4 Mg-ATP, 10 Na-phosphocreatine, 0.4 Na_2_-GTP, 5 QX-314, and 0.1 EGTA; ~ 290 mOsm, pH 7.22. Electrophysiological recordings were collected with a Multiclamp 700B amplifier (Molecular Devices) and a custom data acquisition program in the Igor Pro software (Wavemetrics). mEPSCs and mIPSCs were acquired in voltage-clamp configuration at − 80 mV and 0 mV, respectively, and corrected for a 12-mV junction potential. mEPSCs and mIPSCs were collected at 10 kHz and filtered at 3 kHz. Pipette capacitance was completely compensated, and series resistance was compensated by 50%. Series resistance was < 18 MΩ for all recordings and data were omitted if input resistance changed by ± 15%. Events were analyzed using a deconvolution-based event detection algorithm within Igor Pro [24]. Detectable events were identified using a noise threshold of 3.5× with a minimum amplitude of 2 pA and a 2-ms inter-event interval. Events were subsequently manually screened to confirm appropriate event detection. Event detection code is available at https://benderlab.ucsf.edu/resources. Data were acquired from both sexes and no sex-dependent differences were found. All data were analyzed blind to genotype. Statistical analyses were conducted using the GraphPad Prism 8.4 software. Data are shown as min. to max. box and whisker plots. Each data point (*n*) is indicative of individual neurons. The mean values per cell were compared using the Mann-Whitney test. Cumulative probability distribution of mEPSCs and mIPSCs event intervals were generated per cell and then averaged. Distributions were compared using the Kolmogorov-Smirnov test. A confidence interval of 95% (*P* < 0.05) was required for values to be considered statistically significant.

### Quantification and statistical analysis

All individual data points are shown as well as mean ± SEM. All statistical analyses were performed using the GraphPad Prism 7.0 software. Statistical significance was accepted at the level *p* < 0.05. We used Student’s *t*-test to compare pairs of groups if data were normally distributed (verified using the Lillie test). We examined the changes in synapse numbers from *n* = 2 animals for each genotype. The number of dendrites analyzed is specified in the figure legends. Miniature recording experiments at P70-110 were conducted from *n* = 3 different animals for each age and genotype. The specific *n* for each experiment as well as the post hoc test and the corrected *p* values can be found in the figure legends.

## Results

### Excitatory and inhibitory synapses are reduced in adult *Tbr1* CKO mutants

To evaluate whether *Tbr1* mouse mutants have a stable synaptic deficit, we examined the number of excitatory and inhibitory synapses onto the apical dendrites of adult *Tbr1*^*wildtype*^, *Tbr1*^*layer5CKO*^, and *Tbr1*^*layer6CKO*^. We compared *Tbr1*^*wildtype*^ and *Tbr1*^*layer5CKO*^ neurons within layers 2/3 of the medial prefrontal cortex (mPFCx), and *Tbr1*^*wildtype*^ and *Tbr1*^*layer6CKO*^ neurons within layer 5 in the somatosensory cortex (SSCx) at P181 and P208. Layer 5 and layer 6 projection neurons were tdTomato^+^ through labeling with *Rbp4-cre::tdTomato*^*f/+*^ and *Ntsr1-cre::tdTomato*^*f/+*^, respectively. A functional or mature synapse was defined by the overlap of either presynaptic PSD95 and postsynaptic VGLUT1 (excitatory synapse) or presynaptic Gephyrin/ postsynaptic VGAT (inhibitory synapse) onto the endogenous *tdTomato*^*+*^ structures.

**Fig. 1:**
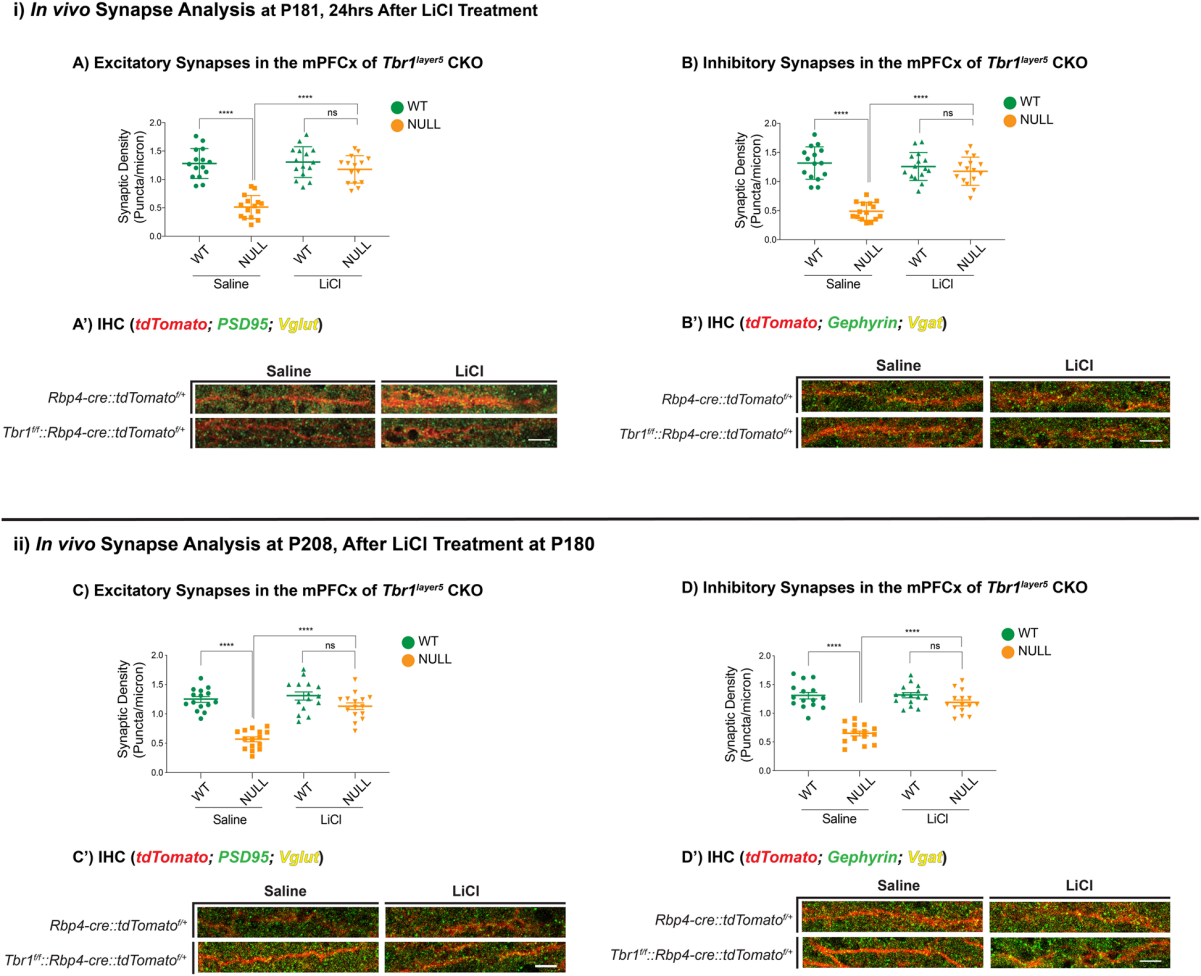
LiCl treatment restores normal synapse numbers in *Tbr1*^*layer5*^ mutant mice. Immunofluorescence (IF) was used to detect excitatory (**A**, **C**) and inhibitory (**B**, **D**) synapses onto dendrites from mPFCx of *Tbr1*^*wildtype*^ (*Rbp4-cre::tdTomato*^*f/+*^; green), *Tbr1*^*layer5*^ CKOs (*Tbr1*^*f/f*^*::Rbp4-cre::tdTomato*^*f/+*^; orange) (*n* = 10 dendrites). Synapses were measured (i) 24 h and (ii) 4 weeks after injection with saline or LiCl at P180. Excitatory synapses were analyzed by VGlut1^+^ boutons and PSD95^+^ clusters co-localizing onto the dendrites from layer 5 neurons of mPFCx of *Tbr1*^*wildtype*^ (green) and *Tbr1*^*layer5CKO*^ (orange) mice 24 h (at P181; A, A’) and 4 weeks (P208; C, C’) after saline and/or LiCl was administered. *Mann-Whitney* ****p < 0.0001, ns = not significant. Inhibitory synaptic density was measured by VGat^+^ boutons and Gephyrin^+^ clusters co-localizing onto dendrites of mPFCx of *Tbr1*^*wildtype*^ (green) and *Tbr1*^*layer5CKO*^ (orange) 24 h (at P181; B, B’) and 4 weeks (P208; D, D’) after saline and/or LiCl was administered. *Mann-Whitney* ****p < 0.0001, ns = not significant. The Fiji ImageJ software was used to process confocal images for quantification. Two-tailed *T*-test with *Mann-Whitney’s* correction was used for pairwise comparisons

We measured the excitatory synaptic density by analyzing VGLUT1^+^ presynaptic terminals that were apposed to dendritic postsynaptic zones (PSD95^+^) using immunofluorescence (IF) and confocal microscopy (Fig. 1A, A’). Inhibitory synaptic density was assessed by counting the overlapping VGAT^+^ presynaptic inhibitory terminals and Gephyrin^+^ dendritic postsynaptic zones on the apical dendrites (Fig. 1B, B’). Excitatory and inhibitory synapse density was decreased 70% and 73% in *Tbr1*^*layer5*^ CKOs, and 74% and 68% in *Tbr1*^*layer6*^ CKOs at P181 (Figs. 1 and S1) and P208 (Figs. 1 and S1), respectively.

We then examined whether lithium treatment could rescue the synaptic deficit in adult *Tbr1*^*layer5*^ and *Tbr1*^*layer6*^ CKOs (Figs. 1 and S1). The control and LiCl treated brains were harvested either at P181 (24 h after injection), or at P208 (4 weeks after injection; Fig. 1). Confocal images of IF from mPFCx (layer 5) and SSCx (layer 6) showed a nearly complete rescue of synaptic densities, 24 h and 4 weeks after treatment (Figs. 1 and S1). Thus, LiCl treatment of the adult *Tbr1*^*layer5*^, and *Tbr1*^*layer6*^ mutant mice rescued both excitatory and inhibitory synaptic deficit (Figs. 1 and S1).

Furthermore, we investigated the long-term efficacy of lithium treatment on rescuing synaptic deficit in *Tbr1*^*layer5*^ and *Tbr1*^*layer6*^ CKOs. To this end, we treated *Tbr1*^*layer5*^ and *Tbr1*^*layer6*^ CKOs at P30 with a single IP injection as before and examined the changes in synaptic density 6 months post-treatment at P210 (Fig. S2). Lithium treatment resulted in a sustained rescue of the synaptic deficit in *Tbr1*^*layer5*^ and *Tbr1*^*layer6*^ CKOs (Fig. S2), though the percent recovery is lower after a long-term treatment with lithium. Thus, providing in vivo evidence that synaptic deficit phenotype persists into adulthood and that efficacy of the LiCl treatment is sufficient to restore normal synapse numbers in adult *Tbr1* CKO mice.

### *Tbr1* CKO mutants have reduced mature dendritic spine density at P180

**Fig. 2:**
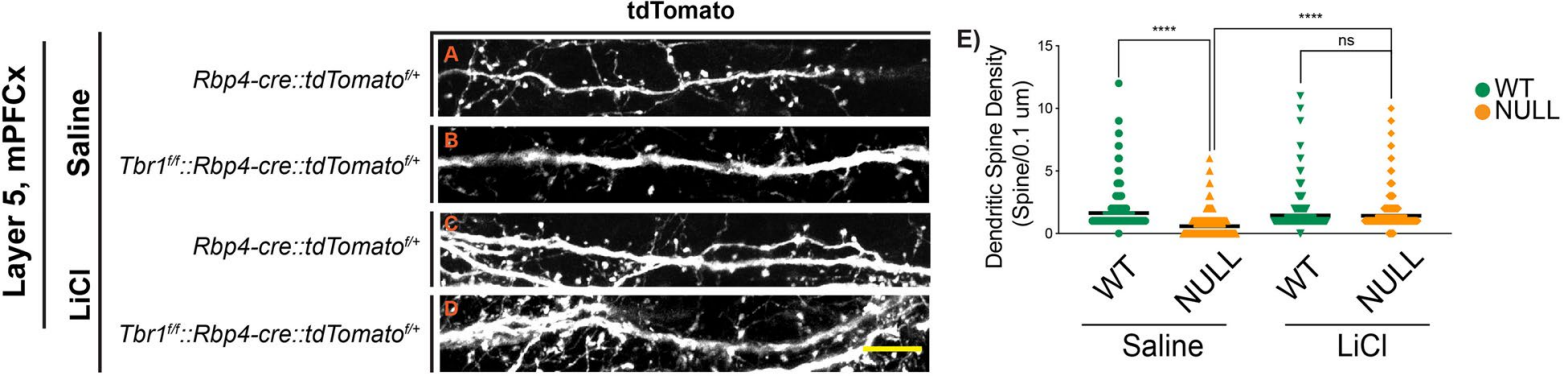
LiCl rescues dendritic spine density deficit in mPFCx of *Tbr1*^*layer5*^ CKOs at P180. *Rbp4-cre::tdTomato*^*f/+*^ allele was used to label the dendrites of layer 5 neurons (**A**–**D**). The monochrome tdTomato signal (white) is shown from apical dendrites of saline-injected (**A**, **B**) and LiCl injected (C, D) *Tbr1* CKOs at P181, 24 h after treatment. The Imaris software (v9.2.1) was used to analyze the dendritic spine density on apical dendrites of *Tbr1*^*layer5*^ wildtype and *Tbr1*^*layer5CKO*^ neurons located within layers 2-4 of mPFCx (**A**–**D**). **E** Quantification of dendritic spine density on apical dendrites of *Tbr1*^*layer5*^ wildtype (green) and *Tbr1*^*layer5CKO*^ neurons (orange) at P181. Spine density was improved 24 h after LiCl treatment (**D**), compared to the saline-injected control animals (**B**). Two-tailed *T*-test with Tukey correction was used for pairwise comparisons. *Mann-Whitney* ****p < 0.0001 (*n* = 10 dendrites), ns = not significant. Scale bar = 2 μm

The synaptic deficits described above prompted us to investigate the state of dendritic spines from the apical dendrites of *Tbr1*^*wildtype*^ and *Tbr1*^*layer5CKO*^ neurons within layers 2/3 in the mPFCx and *Tbr1*^*wildtype*^ and *Tbr1*^*layer6CKO*^ neurons within layer 5 in the SSCx at P180. Layer 5 and layer 6 projection neurons were labeled with *Rbp4-cre::tdTomato*^*f/+*^ and *Ntsr1-cre::tdTomato*^*f/+*^, respectively. We captured × 120 magnification Z-stack images (using × 2 optical zoom) using airyscan confocal microscopy from the apical dendrites of *Tbr1*^*wildtype*^ and *Tbr1*^*layer5CKO*^ neurons (Fig. 2A–D) and *Tbr1*^*wildtype*^ and *Tbr1*^*layer6CKO*^ (Fig. S3A-D) at P180. We used the Imaris software (v9.2.1) to analyze the dendritic spine density and distribution.

The dendritic spine density was reduced in *Tbr1*^*layer5CKO*^ (Fig. 2) and *Tbr1*^*layer6CKO*^ (Fig. S3). Thus, the reduction in the mature dendritic spine density may underlie the synaptic density deficit in *Tbr1* mutants (Figs. 1, 2, S1 and S3). We then tested whether administering LiCl could rescue the reduction in mature spine density in *Tbr1* mutants. We gave a single IP injection of 400 mg/kg LiCl; control animals received a single IP injection of 4 ml/kg saline. LiCl administration rescued the mature dendritic spine density within 24 h in *Tbr1* mutants (Fig. 2E, S3E). The LiCl did not have a clear effect on the density of wild-type dendritic spines (Fig. 2E, S3E). 

### A single dose of LiCl rescues excitatory and inhibitory synapse function in *Tbr1* mutant mice

**Fig. 3:**
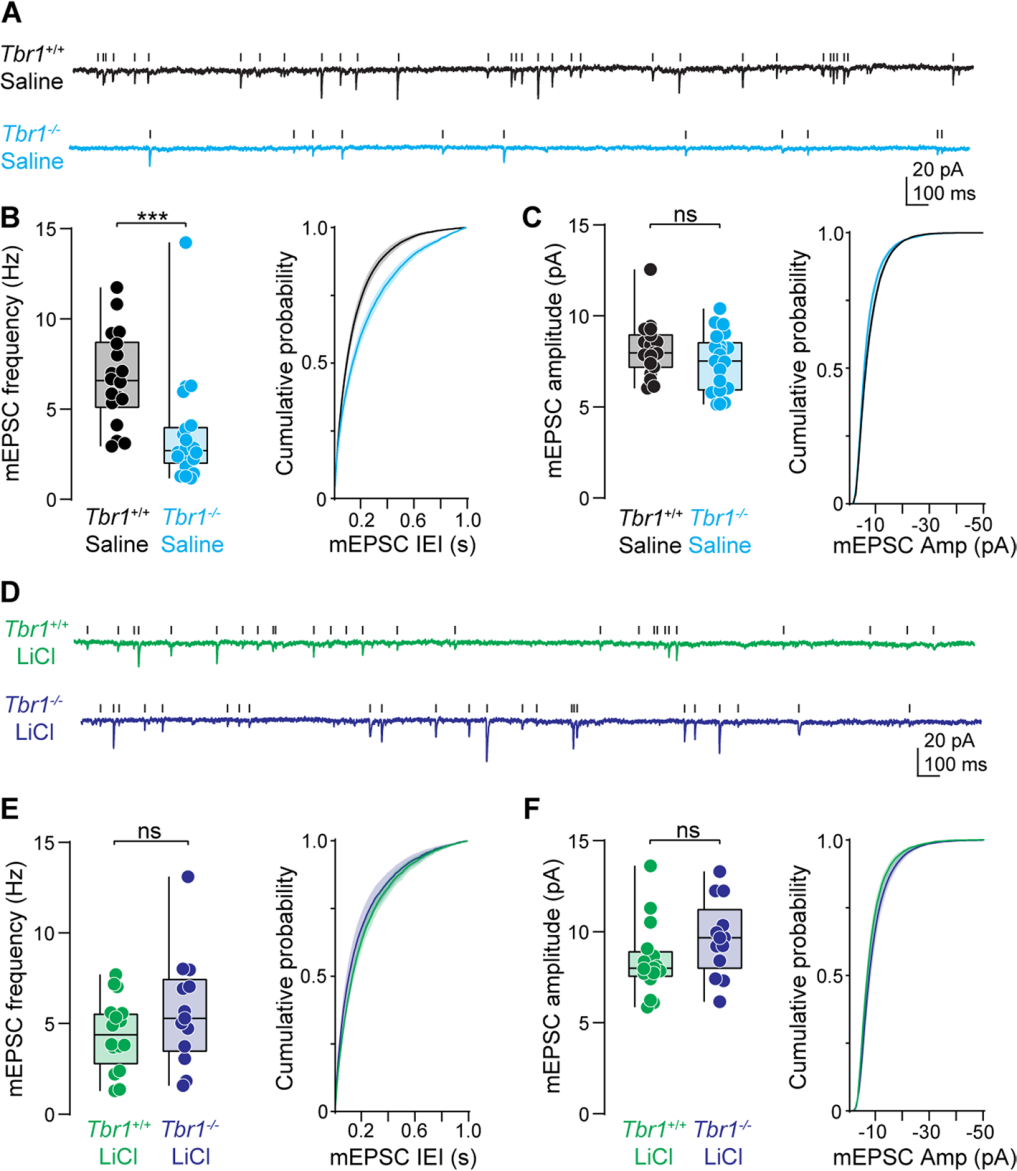
A single LiCl treatment restores excitatory synapse function in *Tbr1*^*layer5*^ CKOs. **A** Representative traces of miniature excitatory postsynaptic currents (mEPSCs) from saline treated (4 ml/kg) P65-106 *Tbr1*^*wildtype*^ (black) and *Tbr1*^*layer5CKO*^ (cyan) layer 5 thick-tufted pyramidal neurons in the medial prefrontal cortex (mPFCx). Scale bars: 20 pA, 100 ms. **B** Left: quantification of mEPSC frequency. *Mann-Whitney* ***p = 0.0001 (*Tbr1*^*wildtype+saline*^: 6.71 ± 0.6 Hz, n = 18, *Tbr1*^*layer5CKO+saline*^ 3.627 ± 0.7 Hz, *n* = 20). Right: cumulative probability distribution of mEPSC inter-event intervals. *Kolmogorov-Smirnov* **p = 0.002. **C** Left: quantification of mEPSC amplitude. *Mann-Whitney p* = 0.32 (*Tbr1*^*wildtype*^: 6.03 ± 0.4 pA, *n* = 18, *Tbr1*^*layer5CKO*^ 5.15 ± 0.4 pA, *n* = 20). Right: cumulative probability distribution of mEPSC amplitude. *Kolmogorov-Smirnov p* = 1.0. **D** Representative traces of mEPSCs from layer 5 neurons of P95-104 *Tbr1*^*wildtype*^ (green) and *Tbr1*^*layer5CKO*^ (purple) treated with 400 mg/kg LiCl at P30. Scale bars: 20 pA, 100 ms. **E** Left: quantification of mEPSC frequency. *Mann-Whitney p* = 0.31 (*Tbr1*^*wildtype+ LiCl*^: 4.43 ± 0.5 Hz, *n* = 16, *Tbr1*^*layer5CKO+ LiCl*^: 5.69 ± 0.9 Hz, *n* = 13). Right: cumulative probability distribution of mEPSC inter-event intervals. *Kolmogorov-Smirnov p* > 0.99. **F** Left: quantification of mEPSC amplitude. *Mann-Whitney p* = 0.09 (*Tbr1*^*wildtype+ LiCl*^: 8.41 ± 0.5 pA, *n* = 16, *Tbr1*^*layer5CKO+ LiCl*^ 9.63 ± 0.6 pA, *n* = 13). Right: cumulative probability distribution of mEPSC inter-event intervals. *Kolmogorov-Smirnov p* > 0.99. Boxplots are min to max show all points

This anatomical reduction in synaptic markers and spine density likely leads to alterations in synapse function. To determine the physiological consequences of *Tbr1* loss-of-function on excitatory synapse function in layer 5 thick-tufted pyramidal neurons in the medial prefrontal cortex (mPFCx), we performed whole-cell voltage-clamp recordings in acute brain slices of *Tbr1*^*wildtype*^ and *Tbr1*^*layer5CKO*^ mice and measured miniature excitatory and inhibitory events. If loss of *Tbr1* within layer 5 neurons reduces synapse numbers, without changing synaptic efficacy (e.g., release probability, postsynaptic receptor density at extant synapses), the miniature synaptic frequency would be reduced without any change in the amplitude of the remaining miniature events. Consistent with this prediction, the frequency of miniature excitatory postsynaptic currents (mEPSCs) was reduced by ~ 45% in P65-106 *Tbr1*^*layer5CKO*^ neurons compared to *Tbr1*^*wildtype*^ (Fig. 3A–B), an extent comparable to the observed reduction in anatomical markers of excitatory synapses (Fig. 1). There was no difference in mEPSC amplitude between *Tbr1*^*layer5CKO*^ neurons compared to *Tbr1*^*wildtype*^ (Fig. 3A, C). This suggests that *Tbr1* loss affects both anatomical and functional aspects of excitatory synapses, perhaps without affecting AMPA receptor density at remaining synapses. Whether postsynaptic *Tbr1* loss affects presynaptic release probability remains unclear.

*Tbr1* is also highly expressed in layer 6 pyramidal neurons within the somatosensory cortex (SSCx) [11, 14]. Therefore, we next investigated the functional implications of *Tbr1* knockout in SSCx layer 6 neurons of adult mice. Recordings of mEPSCs in P70-P110 mice revealed ~ 60% reduction in mEPSC frequency in *Tbr1*^*layer6CKO*^ mice versus *Tbr1*^*wildtype*^ (Fig. S4A-B). In contrast to experiments in layer 5, mEPSC amplitude was reduced in layer 6 (Fig. S4A and C), suggesting that *Tbr1* plays an additional role in regulating either postsynaptic AMPA receptor density or, on average, shifts the location of remaining excitatory synapses to positions more distal to the soma. Despite these differences, the magnitude of reduction in mEPSC frequency is similar between deep-layer pyramidal neurons within the mPFCx and SSCx. Overall, these data indicate that *Tbr1* is critical for normal glutamatergic transmission within these cell types.

Previously, we showed that a single, 400 mg/kg dose of LiCl restored excitatory synapse density on layer 5 pyramidal neurons in *Tbr1*^*layer5CKO*^ mice within 24 h, an effect that is maintained up to 4 weeks post-treatment [15]. Here, we found the morphological components of excitatory synapses were rescued in *Tbr1*^*layer5CKO*^ and *Tbr1*^*layer6CKO*^ mice up to 6 months after one administration of LiCl. But whether a single IP injection of LiCl in *Tbr1* knockout mice restores excitatory synapse function for the long term was unknown. To test this, we first administered LiCl (400 mg/kg) in *Tbr1*^*wildtype*^ and *Tbr1*^*layer5CKO*^ mice at P30 and recorded mEPSCs in acute brain slices at P95-104. Following LiCl treatment, there was no difference in mEPSC frequency or amplitude in *Tbr1*^*layer5CKO*^ mice compared to *Tbr1*^*wildtype*^ littermates up to 8–10 weeks (Fig. 3D–F). We next measured excitatory synapse function in layer 6 SSCx neurons of *Tbr1*^*layer6CKO*^ versus *Tbr1*^*wildtype*^ 8 weeks after LiCl treatment. Neither mEPSC frequency nor amplitude was different in *Tbr1*^*wildtype*^ and *Tbr1*^*layer6CKO*^ layer 6 SSCx pyramidal neurons from P65-95 LiCl treated mice (Fig. S4D-F). Taken together, these data demonstrate that a single dose of LiCl rescues excitatory synapse function in layer 5 and layer 6 pyramidal neurons in the mPFCx and SSCx up to 8 weeks post-treatment.

**Fig. 4:**
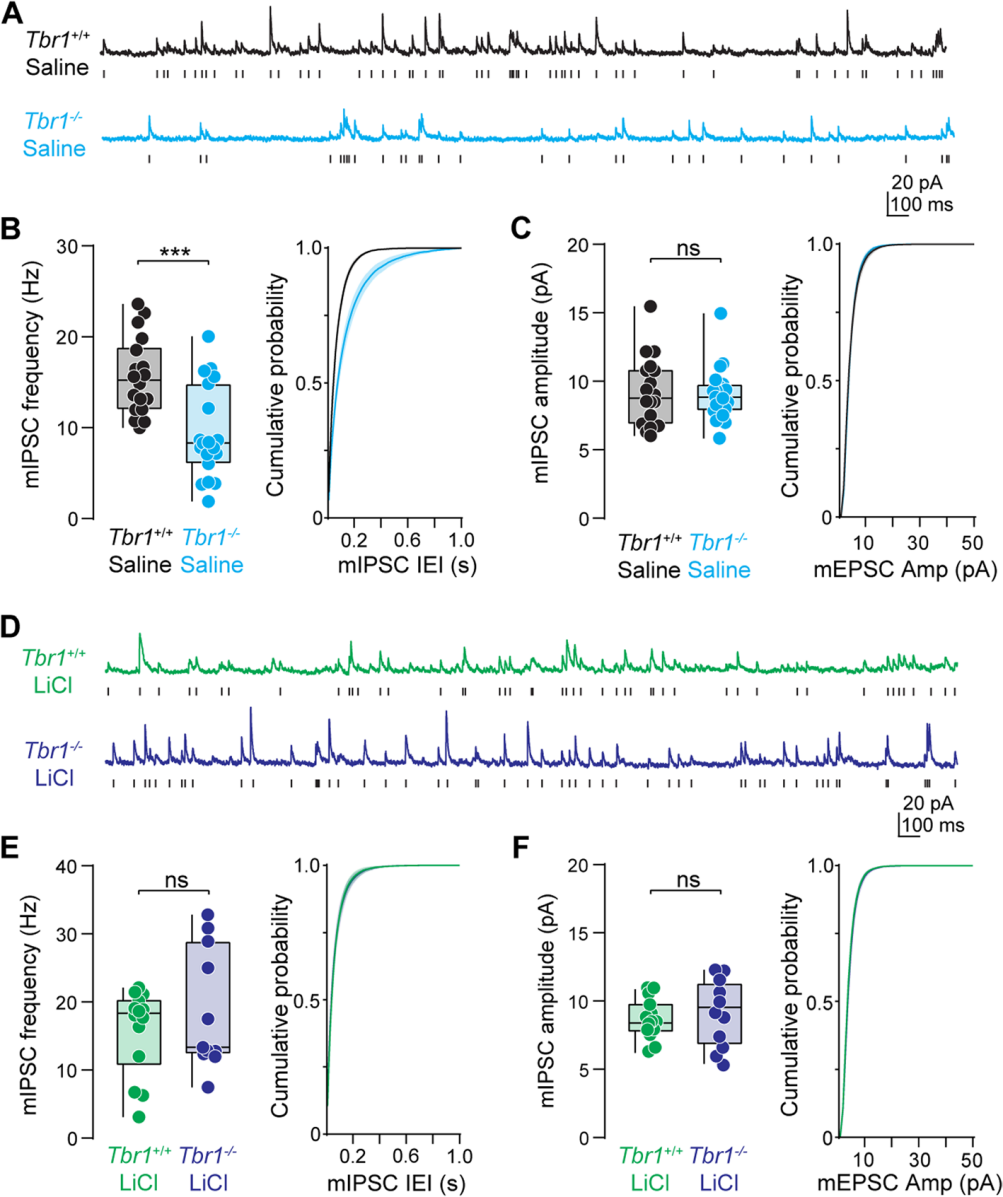
LiCl rescues inhibitory synapse deficits in mPFCx of *Tbr1*^*layer5*^ CKOs. **A** Representative traces of miniature inhibitory postsynaptic currents (mIPSCs) from layer 5 mPFCx neurons of P65-106 *Tbr1*^*wildtype*^ (black) and *Tbr1*^*layer5CKO*^ (cyan) mice following saline administration (4 ml/kg) at P30. Scale bars: 20 pA, 100 ms. **B** Left: quantification of mIPSC frequency. *Mann-Whitney* ***p = 0.0005 (*Tbr1*^*wildtype+saline*^: 15.6 ± 1.0 Hz, *n* = 18, *Tbr1*^*layer5CKO+saline*^ 9.4 ± 1.2 Hz, n = 19). Right: cumulative probability distribution of mIPSC inter-event intervals. *Kolmogorov-Smirnov* ****p < 0.0001. **C** Left: quantification of mIPSC amplitude. *Mann-Whitney p* = 0.32 (*Tbr1*^*wildtype+saline*^: 9.24 ± 0.6 pA, *n* = 18, *Tbr1*^*layer5CKO+saline*^: 9.08 ± 0.5 pA, *n* = 19). Right: cumulative probability distribution of mIPSC amplitude. *Kolmogorov-Smirnov* *p = 0.04. **D** Representative traces of mIPSCs from layer 5 neurons of LiCl (400 mg/kg) treated P95-104 *Tbr1*^*wildtype*^ (green) and *Tbr1*^*layer5CKO*^ (purple) mice. Scale bars: 20 pA, 100 ms. **E** Left: quantification of mIPSC frequency. *Mann-Whitney p* = 0.77 (*Tbr1*^*wildtype+ LiCl*^: 15.84 ± 1.7 Hz, *n* = 14, *Tbr1*^*layer5CKO+ LiCl*^: 18.73 ± 2.7 Hz, *n* = 11). Right: cumulative probability distribution of mIPSC inter-event intervals. *Kolmogorov-Smirnov p* = 0.47. **F** Left: quantification of mIPSC amplitude. *Mann-Whitney p* = 0.57 (*Tbr1*^*wildtype+ LiCl*^: 8.6 ± 0.4 pA, *n* = 14, *Tbr1*^*layer5CKO+ LiCl*^ 9.1 ± 0.8 pA, *n* = 11). Right: cumulative probability distribution of mIPSC inter-event intervals. *Kolmogorov-Smirnov p* = 0.86. Boxplots are min to max show all points

*Tbr1* also plays an essential role in inhibitory synapse development, maintenance, and function [16]. We found that *Tbr1*^*layer5CKO*^ and *Tbr1*^*layer6CKO*^ mice have a lower inhibitory synaptic density at P180 compared to *Tbr1*^*wildtype*^ littermates. Thus, we predicted that fewer inhibitory synapses onto layer 5 and layer 6 pyramidal neurons in *Tbr1* knockout mice would result in reduced frequency of miniature inhibitory postsynaptic currents (mIPSC). To test this, we recorded mIPSCs from layer 5 thick-tufted pyramidal neurons in *Tbr1*^*layer5CKO*^ mice and *Tbr1*^*wildtype*^ controls. mIPSC frequency was significantly reduced in *Tbr1*^*layer5CKO*^ neurons compared to *Tbr1*^*wildtype*^ with no difference in mIPSC amplitude (Fig. 4A–C).

Next, we recorded mIPSCs from layer 6 pyramidal neurons in the SSCx from *Tbr1*^*layer6CKO*^ mice and *Tbr1*^*wildtype*^ littermates. We found similar trends in both the frequency and amplitude of mIPSCs in *Tbr1*^*layer6CKO*^ neurons within the SSCx as observed in *Tbr1*^*layer5CKO*^ neurons within the mPFCx; however, these differences were not statistically significant (Fig. S5A-C). We next wanted to evaluate the long-term efficacy of LiCl treatment on rescuing inhibitory synaptic deficits in *Tbr1*^*layer5CKO*^ and *Tbr1*^*layer6CKO*^ mice. A single dose of LiCl (400 mg/kg) administered at P30 restored the mIPSC frequency deficits in *Tbr1*^*layer5CKO*^ pyramidal neurons in P95-104 mice. LiCl treatment of *Tbr1*^*layer6CKO*^ mice resulted in no difference in mIPSC frequency or amplitude compared to *Tbr1*^*wildtype*^ treated animals (Fig. S5D-F). Thus, a single 400 mg/kg dose of LiCl results in long-term restoration of excitatory and inhibitory synaptic transmission deficits following the loss of *Tbr1* in layer 5 pyramidal neurons of the mPFCx and restores excitatory synaptic deficits in layer 6 pyramidal neurons within the SSCx.

## Discussion and conclusions

### *Tbr1* promotes synaptic numbers and function in adult mouse mPFCx

*Tbr1* is expressed in telencephalic post-mitotic excitatory neurons; its expression is well described in the mouse where it is detected in the neocortex, hippocampus, entorhinal cortex, pallial amygdala, piriform cortex, olfactory bulb, subplate, and Cajal-Retzius neurons [10, 11]. *Tbr1* is best known for its expression and function in layer 6, where it is required to initiate and then maintain layer 6 identity by repressing markers of layer 5 identity [12, 16]. Previously, we demonstrated that *Tbr1* promotes dendritic spine maturation and synaptogenesis in the excitatory neurons of mouse cortical layers 5 and 6 during neonatal and adolescent developmental stages [15, 16]; a phenotype that potentially links *Tbr1*’s function to neuropsychiatric disorders such as ASD. Furthermore, we interrogated these phenotypes in mouse mPFCx, a cortical region with critical functions in cognitive and affective processing.

Towards evaluating whether the LiCl rescue could be relevant for treatment of human *TBR1* mutant patients, we examined whether *Tbr1* is required to maintain dendritic spine maturation and synaptogenesis in adulthood (P180 and P208). We discovered that the adult *Tbr1*^*layer5*^ and *Tbr1*^*layer6*^ CKO mutants have reduced dendritic spine and synaptic densities. Furthermore, adult *Tbr1* CKO mutants have reduced synaptic transmission as demonstrated by the reduction in the frequency of mEPSCs and mIPSCs. Thus, *Tbr1* is required for the long-term synaptic development, maintenance, and function of the excitatory neurons of cortical layers 5 and 6.

### LiCl rescues dendritic spine maturation and synaptic transmission in *Tbr1* mutants

WNT signaling is well-known to control synapse development [25, 26]. WNT signaling is essential in postsynaptic differentiation of excitatory synapses by recruiting NMDA receptors via promoting PSD95 clustering and local activation of CaMKII within dendritic spines [27]. Furthermore, the *Wnt7b* activation of the canonical WNT pathway has been linked to increased presynaptic inputs in the developing hippocampus [25, 28, 29]. Previously, we showed that LiCl, an FDA-approved drug with multiple effects including acting as a WNT signaling agonist, rescued defects in dendritic spine maturation and reduced synapse numbers in the adolescent *Tbr1* mutants [15, 16]. Here, we examined the efficacy and long-term potency of LiCl treatment in rescuing the dendritic spine and synaptic defects in the adult *Tbr1* mutants. We used two different approaches: (1) administering a single IP LiCl injection at 6 months (P180) and examining the effect 4 weeks post-injection and (2) administering a single IP LiCl injection at P30 and examining the effect 6 months post-injection.

LiCl rapidly (within 24 h) promoted the maturation of dendritic spines in *Tbr1*^*layer5*^ and *Tbr1*^*layer6*^ CKO neurons in cortical layers 5 and 6, respectively. Furthermore, LiCl rescued excitatory and inhibitory synapse numbers within 24 h. Remarkably, a single dose of LiCl at P30 led to a sustained rescue of synaptic density, measured 6 months after treatment. Furthermore, LiCl treatment rescued the reduction in the mEPSCs and mIPSCs. Thus, LiCl treatment led to a long-term restoration of excitatory and inhibitory synaptic transmission deficits following the loss of *Tbr1* in layer 5 and 6 pyramidal neurons.

### LiCl as a potential therapy for neurodevelopmental disorders with reduced synapse transmission

ASD is a neurodevelopmental disorder and patients typically display symptoms before the age of three [30]. One of the key questions in autism research is whether the pathology is reversible, and if so, at what ages can it be ameliorated. Currently, there are no treatments for ASD that address its core biological defects; we suggest that reduced synapse numbers may be one of these core defects in at least a subset of patients, such as those who have a function altering *TBR1* mutation. In addition to *TBR1*, mutations in *SHANK3* (another high confidence ASD-risk gene) have been shown to result in synaptic defects and autistic-like behaviors including anxiety, social interaction deficits, and repetitive behavior [31–33]. Thus, it has been postulated that synapse numbers (connectivity) and synaptic function are points of convergence in ASD pathophysiology [34].

The ability to restore synapse numbers and transmission following LiCl administration in the adult *Tbr1* mutant mice provides an insight to a possible human therapy, especially given that LiCl is an FDA-approved drug with well-described side effects [35]. Even though our current work suggests the applicability of LiCl for ASD patients with *TBR1* mutations, LiCl could be considered for other ASD syndromes. This hypothesis is further strengthened by a clinical case report where two ASD patients (a 21-year-old male and a 17-year-old female) with mutations in *SHANK3* underwent lithium treatment. Lithium treatment in these two patients improved the clinical regression, stabilized behavioral abnormalities, and restored brain function to their pre-catatonia levels without reported adverse side effects [36].

Remarkable properties of LiCl in treating mouse *Tbr1* mutants are (1) its ability to rapidly (24 h) promote dendritic spine maturation and synaptogenesis and (2) the 6-month persistence of its action after only a single LiCl single dose. We suggest that the *Tbr1* mutants may have immature synapses that are poised to mature once they receive the appropriate signal (perhaps a WNT agonist) [15] and once achieved, the synapses are maintained and function at a reasonable level. Thus, LiCl may have some promise as a therapy for ASD patients with either mutations in *TBR1* or for cases due to other genetic or non-genetic etiologies that have a similar block in synaptic development.

## Supplementary Information

**Additional file 1.**

## Data Availability

Data and materials that were used in this study are available upon request from the lead contact.

## Abbreviations

ASD: Autism spectrum disorder
mEPSC: Miniature excitatory post-synaptic current
mIPSC: Miniature inhibitory post-synaptic current
CKO: Conditional nock-out
mPFCx: Medial prefrontal cortex
SSCx: Somatosensory cortex
IP: Intraperitoneal injection
