# Supplementary material for: LiCl treatment leads to long-term restoration of spine maturation and synaptogenesis in adult Tbr1 mutants
Source: J Neurodev Disord. 2022 Feb 5;14:11. doi: 10.1186/s11689-022-09421-5 (PMC8903688; doi:10.1186/s11689-022-09421-5)
Supplement: Supplementary file 1 — Additional file 1. [file 11689_2022_9421_MOESM1_ESM.pdf]

Figure S1: LiCl Treatment Rescues Synaptic Deficit in SSCx of *Tbr1^{layer6}* mutant mice.

Immunofluorescence (IF) was used to detect excitatory **(A, C)** and inhibitory **(B, D)** synapses onto dendrites from SSCx of *Tbr1^{wildtype}* (*Ntsr1-cre::tdTomato$^{f/+}$*; blue), *Tbr1^{layer6}* CKOs (*Tbr1$^{f/f}$::Ntsr1-cre::tdTomato$^{f/+}$*; red) (n=10 dendrites). Synapses were measured **(i)** 24 hrs and **(ii)** 4 weeks after injection with saline or LiCl at P180.
Excitatory synapses were analyzed by VGlut1^{+} boutons and PSD95^{+} clusters co-localizing onto the dendrites from layer 6 neurons of SSCx of *Tbr1^{wildtype}* (red) and *Tbr1^{layer6CKO}* (blue) mice 24 hrs (at P181; A) and 4 weeks (P208; C) after saline and/or LiCl was administered. *Mann-Whitney* ****$p < 0.0001$, ns = not significant.
Inhibitory synaptic density was measured by VGat^{+} boutons and Gephyrin^{+} clusters co-localizing onto dendrites of SSCx of *Tbr1^{wildtype}* (red) and *Tbr1^{layer6CKO}* mice (blue), 24 hrs (at P181; B) and 4 weeks (P208; D) after saline and/or LiCl was administered. ImageJ software was used to process confocal images for quantification. Two-tailed T-test with *Mann-Whitney* correction was used for pairwise comparisons. *Mann-Whitney* ****$p < 0.0001$, ns = not significant.

i) *In vivo* Synapse Analysis at P181, 24hrs After LiCl Treatment

A) Excitatory Synapses in the SSCx of *Tbr1*^{layer6} CKO

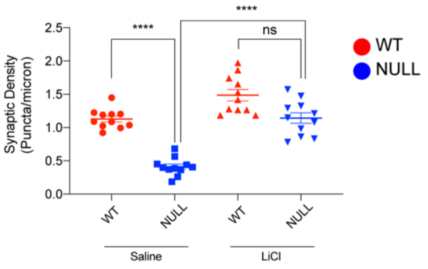

B) Inhibitory Synapses in the SSCx of *Tbr1*^{layer6} CKO

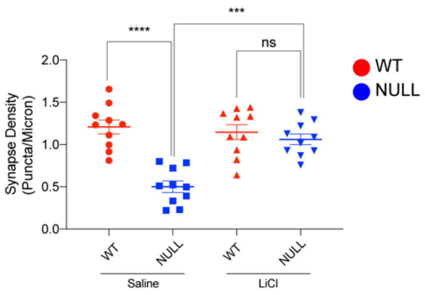

ii) *In vivo* Synapse Analysis at P208, After LiCl Treatment at P180

C) Excitatory Synapses in the SSCx of *Tbr1*^{layer6} CKO

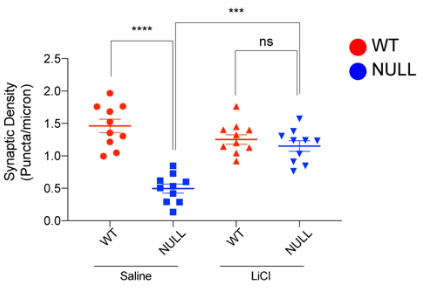

D) Inhibitory Synapses in the SSCx of *Tbr1*^{layer6} CKO

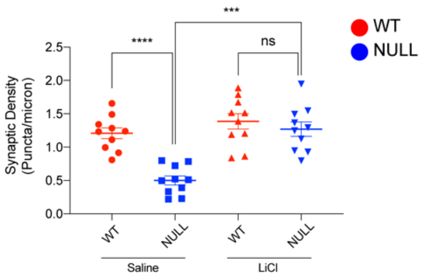

Figure S2: LiCl Treatment Results in long-term Restoration of Synaptic Deficit in *Tbr1* Mutants.

Immunofluorescence (IF) was used to detect excitatory **(i)** and inhibitory **(ii)** synapses onto dendrites from mPFCx of $Tbr1^{wildtype}$ ($Rbp4\text{-}cre::tdTomato^{f/+}$; green), $Tbr1^{layer5}$ CKOs ($Tbr1^{f/f}::Rbp4\text{-}cre::tdTomato^{f/+}$; orange) and dendrites from SSCx of $Tbr1^{wildtype}$ ($Ntsr1\text{-}cre::tdTomato^{f/+}$; red), $Tbr1^{layer6}$ CKOs ($Tbr1^{f/f}::Ntsr1\text{-}cre::tdTomato^{f/+}$; blue) (n=10 dendrites). Synapses were measured 6 months after injection with saline or LiCl at P30.

(i) Excitatory synapses were analyzed by $VGlut1^+$ boutons and $PSD95^+$ clusters co-localizing onto the dendrites from (A) layer 5 neurons of mPFCx of $Tbr1^{wildtype}$ (green) and $Tbr1^{layer5CKO}$ (orange), (B) layer 6 neurons of SSCx of $Tbr1^{wildtype}$ (red) and $Tbr1^{layer6CKO}$ (blue) mice 6 months after saline and/or LiCl was administered. *Mann-Whitney* ****$p < 0.0001$, ns = not significant.

(ii) Inhibitory synaptic density was measured by $VGat^+$ boutons and $Gephyrin^+$ clusters co-localizing onto dendrites of (C) mPFCx of $Tbr1^{wildtype}$ (green) and $Tbr1^{layer5CKO}$ (orange) and (D) SSCx of $Tbr1^{wildtype}$ (red) and $Tbr1^{layer6CKO}$ mice (blue), 6 months after saline and/or LiCl was administered. Fiji ImageJ software was used to process confocal images for quantification. Two-tailed T-test with *Mann-Whitney* correction was used for pairwise comparisons. *Mann-Whitney* ****$p < 0.0001$, ns = not significant.

i) *In vivo* Excitatory Synapse Analysis of *Tbr1* Conditional Mutant at P210, 6 months After LiCl Treatment

A) mPFCx of *Tbr1*^{layer5} CKO

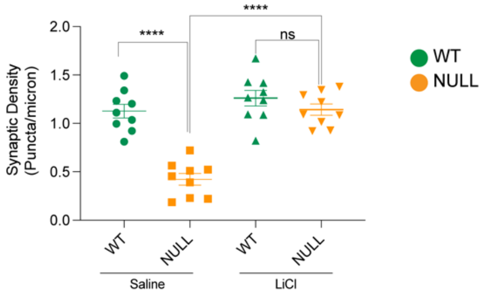

B) SSCx of *Tbr1*^{layer6} CKO

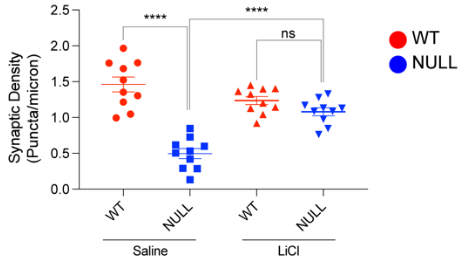

ii) *In vivo* Inhibitory Synapse Analysis of *Tbr1* Conditional Mutant at P210, 6 months After LiCl Treatment

C) mPFCx of *Tbr1*^{layer5} CKO

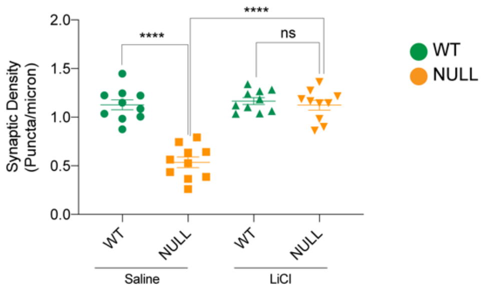

D) SSCx of *Tbr1*^{layer6} CKO

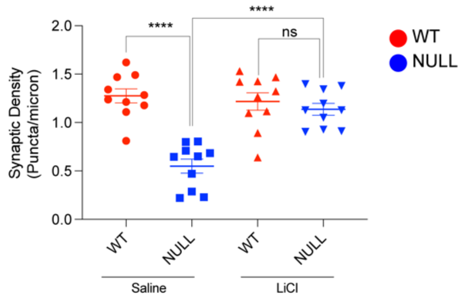

Figure S3: LiCl Restores Dendritic Spine Density Deficit in SSCx of *Tbr1*^{layer6} CKOs at P180.

Ntsr1-cre::tdTomato$^{f/+}$ alleles was used to label the dendrites of layer 6 neurons (A-D). The monochrome tdTomato signal (white) is shown from apical dendrites of saline-injected (A, B) and LiCl injected (C, D) *Tbr1* CKOs at P181. Imaris software (v9.2.1) was used to analyze the dendritic spine density on apical dendrites of *Tbr1*^{layer6} wildtype and *Tbr1*^{layer6CKO} neurons located within layer 5 of SSCx (A-D). Changes in the dendritic spine density were examined at P181 (A-D), 24 hrs after LiCl treatment. (E) Quantification of mature dendritic spines on apical dendrites of *Tbr1*^{layer6} wildtype and *Tbr1*^{layer6CKO} neurons at P181, 24 hrs after injection with saline (control) or LiCl. Spine density was improved 24 hrs after LiCl treatment (D), compared to the saline-injected control animals (B). Two-tailed T-test with Tukey correction was used for pairwise comparisons. *Mann-Whitney* ****$p < 0.0001$ (n = 10 dendrites), ns = not significant. Scale bar = 2 μm.

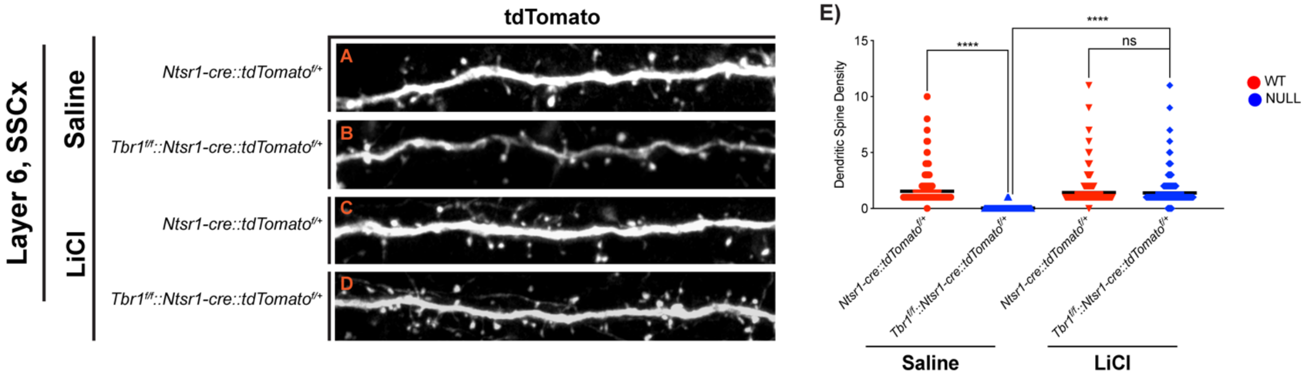

tdTomato
Layer 6, SSCx
Saline
LiCl
Ntsr1-cre::tdTomato^f/+
Tbr1^f/f::Ntsr1-cre::tdTomato^f/+
Ntsr1-cre::tdTomato^f/+
Tbr1^f/f::Ntsr1-cre::tdTomato^f/+
A
B
C
D
E)
Dendritic Spine Density
15
10
5
0
****
****
ns
WT
NULL
Ntsr1-cre::tdTomato^f/+
Tbr1^f/f::Ntsr1-cre::tdTomato^f/+
Ntsr1-cre::tdTomato^f/+
Tbr1^f/f::Ntsr1-cre::tdTomato^f/+
Saline
LiCl

Figure S4: A single LiCl dose restores excitatory synaptic function in SSCx of *Tbr1*^{layer6} CKOs.

(**A**) Representative traces of miniature excitatory postsynaptic currents (mEPSCs) from P58-95 *Tbr1*^{wildtype} (black) and *Tbr1*^{layer6CKO} (cyan) SSCx layer 6 neurons following saline administration. Scale bars: 20 pA, 100 ms. (**B**) Left: quantification of mEPSC frequency. *Mann-Whitney* ***p = 0.0002 (*Tbr1*$^{wildtype+saline}$: 7.33 ± 1.2 Hz, n = 11, *Tbr1*$^{layer6CKO+saline}$ 2.8 ± 0.4 Hz, n = 8). Right: cumulative probability distribution of mEPSC inter-event intervals. *Kolmogorov-Smirnov* *p = 0.016. (**C**) Left: quantification of mEPSC amplitude. *Mann-Whitney* **p = 0.001 (*Tbr1*^{wildtype}: 7.6 ± 0.4 pA, n = 11, *Tbr1*^{layer6CKO} 5.5 ± 0.3 pA, n = 8). Right: cumulative probability distribution of mEPSC amplitude. *Kolmogorov-Smirnov* p = 0.27. (**D**) Representative traces of mEPSCs from layer 6 SSCx neurons from P58-95 *Tbr1*^{wildtype} (green) and *Tbr1*^{layer6CKO} (purple) treated with 400 mg/kg LiCl at P30. Scale bars: 20 pA, 100 ms. (**E**) Left: quantification of mEPSC frequency. *Mann-Whitney* p = 0.1 (*Tbr1*$^{wildtype+ LiCl}$: 4.1 ± 1.0 Hz, n = 11, *Tbr1*$^{layer6CKO+ LiCl}$ 4.4 ± 0.5 Hz, n = 14). Right: cumulative probability distribution of mEPSC inter-event intervals. *Kolmogorov-Smirnov* p = 0.7. (**F**) Left: quantification of mEPSC amplitude. *Mann-Whitney* p = 0.09 (*Tbr1*$^{wildtype+ LiCl}$: 6.6 ± 0.6 pA, n = 11, *Tbr1*$^{layer6CKO+ LiCl}$ 7.5 ± 0.3 pA, n = 14). Right: cumulative probability distribution of mEPSC inter-event intervals. *Kolmogorov-Smirnov* p = 0.54. Boxplots are min to max show all points.

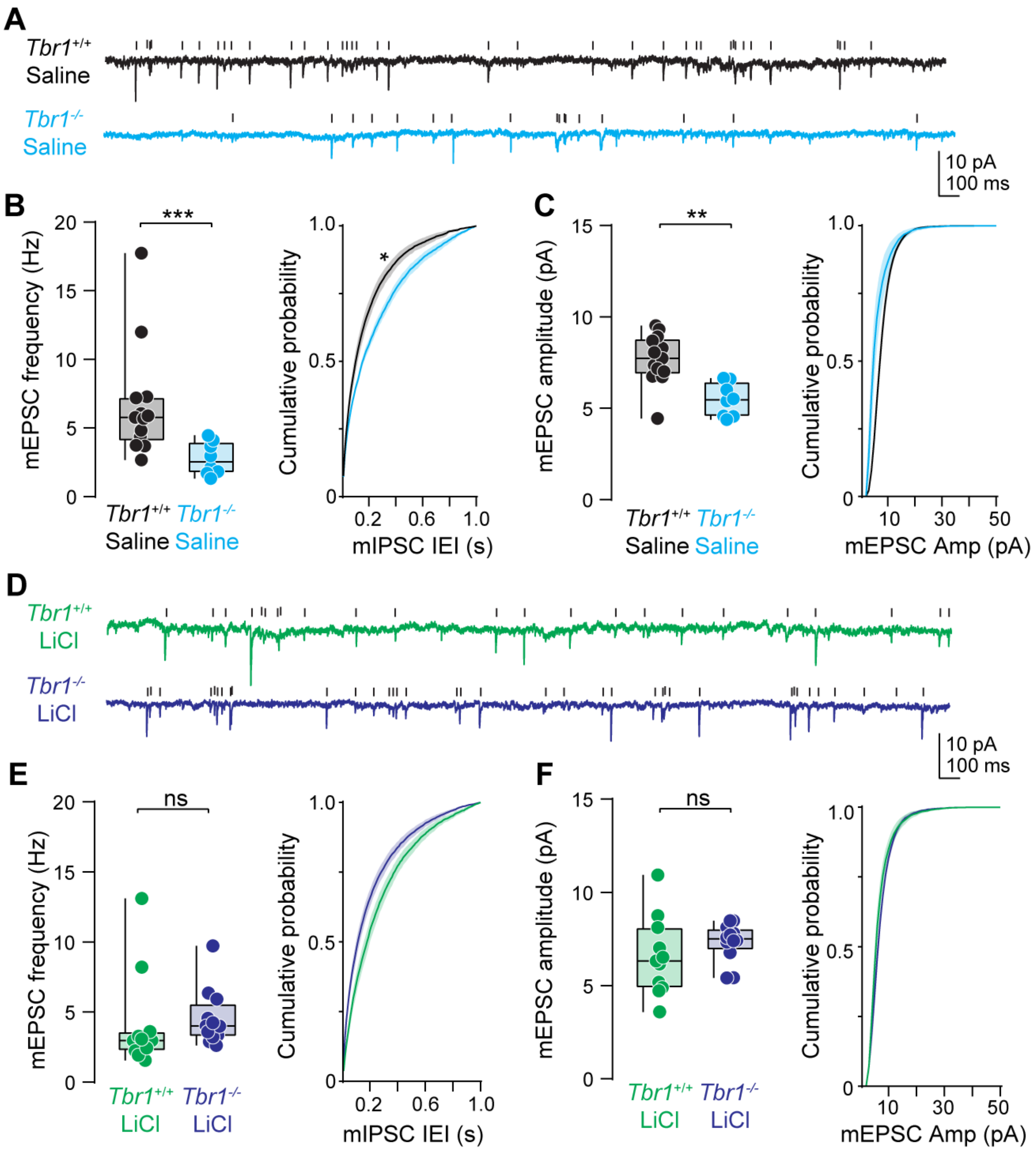

A
Tbr1+/+ Saline
Tbr1-/- Saline
10 pA
100 ms
B
***
mEPSC frequency (Hz)
20
15
10
5
0
Tbr1+/+ Tbr1-/-
Saline Saline
Cumulative probability
1.0
0.5
0
*
0.2
0.6
1.0
mIPSC IEI (s)
C
**
mEPSC amplitude (pA)
15
10
5
0
Tbr1+/+ Tbr1-/-
Saline Saline
Cumulative probability
10
30
50
mEPSC Amp (pA)
D
Tbr1+/+ LiCl
Tbr1-/- LiCl
10 pA
100 ms
E
ns
mEPSC frequency (Hz)
Tbr1+/+ Tbr1-/-
LiCl LiCl
Cumulative probability
mIPSC IEI (s)
F
ns
mEPSC amplitude (pA)
Tbr1+/+ Tbr1-/-
LiCl LiCl
Cumulative probability
mEPSC Amp (pA)

Figure S5: LiCl treatment rescues inhibitory synaptic deficits in SSCx of *Tbr1*^{layer6} CKOs.

(**A**) Representative traces of miniature inhibitory postsynaptic currents (mIPSCs) from SSCx layer 6 pyramidal neurons from saline treated P58-95 *Tbr1*^{wildtype} (black) and *Tbr1*^{layer6CKO} (cyan) mice. Scale bars: 20 pA, 100 ms. (**B**) Left: quantification of mIPSC frequency from *Tbr1*^{wildtype} (black) and *Tbr1*^{layer6CKO} (cyan) neurons. *Mann-Whitney* p = 0.28 (*Tbr1*$^{wildtype+saline}$: 14.8 ± 3.8 Hz, n = 9, *Tbr1*$^{layer6CKO+saline}$ 8.7 ± 2.4 Hz, n = 8). Right: cumulative probability distribution of mIPSC inter-event intervals. *Kolmogorov-Smirnov* *p = 0.01. (**C**) Left: quantification of mIPSC amplitude. *Mann-Whitney* p = 0.74 (*Tbr1*$^{wildtype+saline}$: 12.2 ± 1.3 pA, n = 9, *Tbr1*$^{layer6CKO+saline}$ 11.1 ± 0.7 pA, n = 8). Right: cumulative probability distribution of mIPSC amplitude. *Kolmogorov-Smirnov* *p = 0.012. (**D**) Representative traces of mIPSCs from layer 6 SSCx neurons from LiCl (400 mg/kg) treated P58-95 *Tbr1*^{wildtype} (green) and *Tbr1*^{layer6CKO} (purple) mice. Scale bars: 20 pA, 100 ms. (**E**) Left: quantification of mIPSC frequency. *Mann-Whitney* p = 0.2 (*Tbr1*$^{wildtype+ LiCl}$: 9.5 ± 2.6 Hz, n = 8, *Tbr1*$^{layer6CKO+ LiCl}$ 12.8 ± 3.1 Hz, n = 9). Right: cumulative probability distribution of mIPSC inter-event intervals. *Kolmogorov-Smirnov* *p = 0.01. (**F**) Left: quantification of mIPSC amplitude. *Mann-Whitney* p = 0.96 (*Tbr1*$^{wildtype+ LiCl}$: 12.3 ± 0.6 pA, n = 8, *Tbr1*$^{layer6CKO+ LiCl}$ 12.6 ± 0.6 pA, n = 9). Right: cumulative probability distribution of mIPSC inter-event intervals. *Kolmogorov-Smirnov* p = 0.99. Boxplots are min to max show all points.

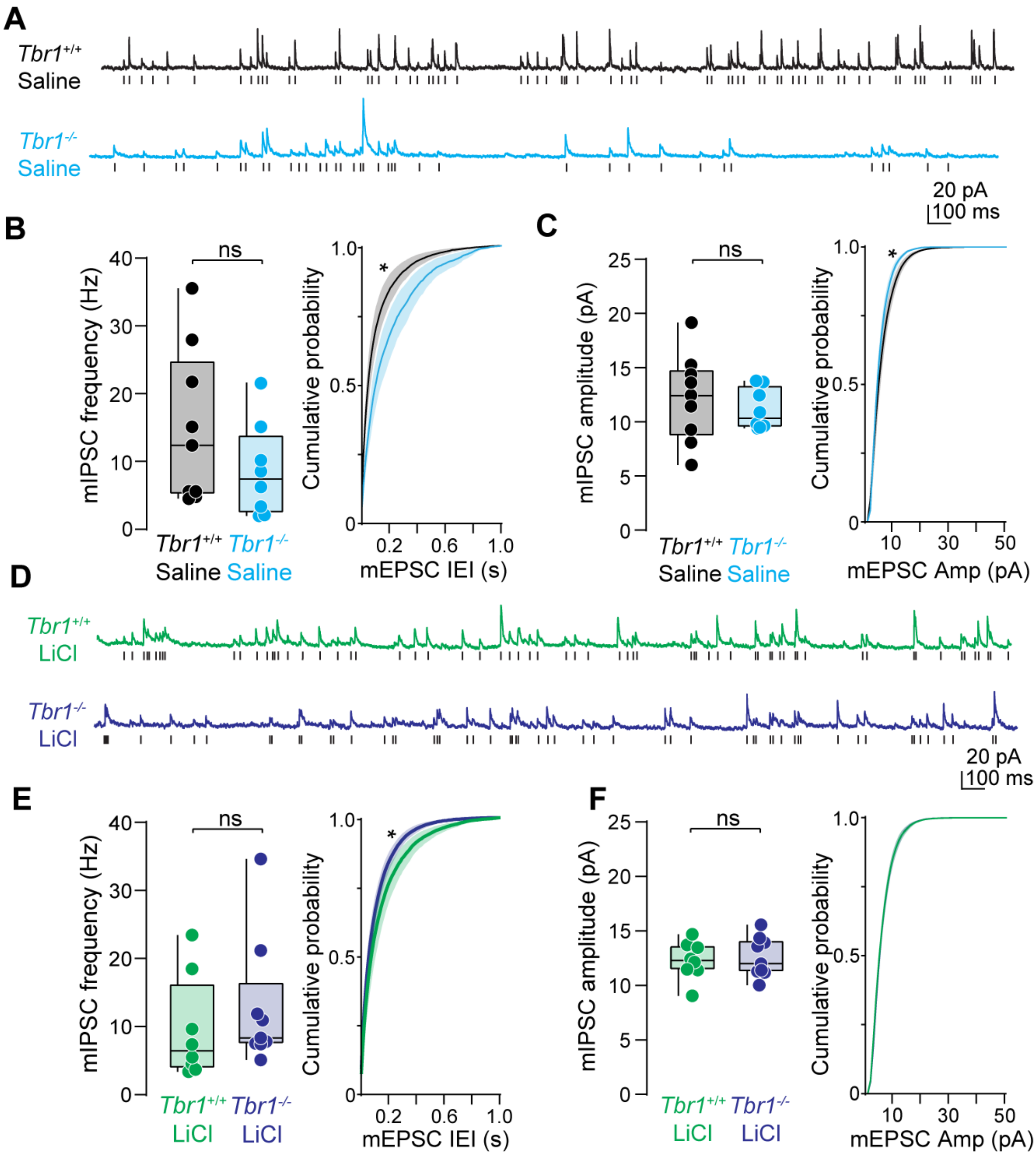
A
Tbr1+/+
Saline
Tbr1-/-
Saline
20 pA
100 ms
B
mIPSC frequency (Hz)
ns
Tbr1+/+ Tbr1-/-
Saline Saline
Cumulative probability
mEPSC IEI (s)
C
mIPSC amplitude (pA)
ns
Tbr1+/+ Tbr1-/-
Saline Saline
Cumulative probability
mEPSC Amp (pA)
D
Tbr1+/+
LiCl
Tbr1-/-
LiCl
20 pA
100 ms
E
mIPSC frequency (Hz)
ns
Tbr1+/+ Tbr1-/-
LiCl LiCl
Cumulative probability
mEPSC IEI (s)
F
mIPSC amplitude (pA)
ns
Tbr1+/+ Tbr1-/-
LiCl LiCl
Cumulative probability
mEPSC Amp (pA)
